# Use of a gene score of multiple low-modest effect size variants can predict the risk of obesity better than the individual SNPs

**DOI:** 10.1186/s12944-018-0806-5

**Published:** 2018-07-18

**Authors:** Shabana, Saleem Ullah Shahid, Shahida Hasnain

**Affiliations:** Department of Microbiology and Molecular Genetics, University of the Punjab, Lahore, Pakistan

**Keywords:** Obesity, Gene score, Polygenic, Risk variant

## Abstract

**Background:**

Obesity is a complex disorder, the development of which is modulated by a multitude of environmental, behavioral and genetic factors. The common forms of obesity are polygenic in nature which means that many variants in the same or different genes act synergistically and affect the body weight quantitatively. The aim of the current study was to use information from many common variants previously identified to affect body weight to construct a gene score and observe whether it improves the associations observed. The SNPs selected were G2548A in leptin (*LEP*) gene, Gln223Arg in leptin receptor (*LEPR*) gene, Ala54Thr in fatty acid binding protein 2 (*FABP2*) gene, rs1121980 in fat mass and obesity associated (*FTO*) gene, rs3923113 in Growth Factor Receptor Bound Protein 14 (*GRB14)*, rs16861329 in Beta-galactoside alpha-2,6-sialyltransferase 1 (*ST6GAL1)*, rs1802295 in Vacuolar protein sorting-associated protein 26A (*VPS26A)*, rs7178572 in high mobility group 20A (*HMG20A)*, rs2028299 in adaptor-related protein complex 3 (*AP3S2)*, and rs4812829 in Hepatocyte Nuclear Factor 4 Alpha (*HNF4A*).

**Methods:**

A total of 475 subjects were genotyped for the selected SNPs in different genes using different genotyping techniques. The study subjects’ age, weight, height, BMI, waist and hip circumference, serum total cholesterol, triglycerides, LDL and HDL were measured. A summation term, genetic risk score (GRS), was calculated using SPSS.

**Results:**

The results showed a significantly higher mean gene score in obese cases than in non-obese controls (9.1 ± 2.26 vs 8.35 ± 2.07, *p* = 2 × 10^− 4^). Among the traits tested for association, gene score appeared to significantly affect BMI, waist circumference, and all lipid traits.

**Conclusion:**

In conclusion, the use of gene score is a better way to calculate the overall genetic risk from common variants rather than individual risk variants.

**Electronic supplementary material:**

The online version of this article (10.1186/s12944-018-0806-5) contains supplementary material, which is available to authorized users.

## Background

Obesity is a defined as the excess of body fat. It is a complex disorder, influenced by a number of genetic and environmental factors. There has been a dramatic increase in the number of overweight and obese individuals, both children and adults, globally [1]. Pakistan with a total population of 184.35 million in 2012–13 is the 6th most populous country of the world [2]. According to the Global Burden of Disease Study, Pakistan ranked 9th out of 188 countries in terms of obesity [3].

Traditionally, before the advent of high throughput genotyping methodologies, the contribution of genes to the risk of development of disease was recognized through the increased risk of disease in the proband’s relatives. The genetic component was then expressed as heritability estimates or variance components. However, rapid developments of high-throughput genetic technologies have led to the genome-wide association studies (GWAS) [4]. The GWASs analyze common variations by genotyping of a large number of SNPs (~ 0.5–1 million) in a case control study design. The results are then used to determine which of these SNPs reach genome wide significance level with the outcome (mostly the disease) [5]. One problem with common variants is their small effect sizes (*the contribution of a SNP to the genetic variance of a trait*) accounting for a small fraction of variance in the disease risk. Familial clustering of complex diseases suggests that the heritable risk factors are of large effect sizes therefore a GWAS is unable to detect such variants because of a very low frequency. The situation is further complicatede due to epistatic effects resulting from the interaction of variants in different genes. The epistatic effects thus confound the search for new loci because their probability is the product of probabilities of low frequency individual variants [6].

The genetics of complex disease is inherently based on statistical methods because the phenomenon (e.g., obesity) being a complex disorder is itself probabilistic by definition. In order to interpret meaningful results from a dataset, various statistical methods are needed. The commonly used statistical procedures include use of risk prediction algorithms (relative risk, odds ratio), family analyses (liability threshold models) and regression methods (linear/logistic regression) [7]. These methods are based on assumptions which can be very different and even incompatible. In GWASs, the inclusion of a large number of SNPs leads to more accurate gene identification in theory because it is based on the frequency of individual risk alleles. However, this theoretical advantage is reduced either by multiple testing correction (due to the inclusion of many SNPs), or by the increased degrees of freedom. The use of a weighted score test (WST) or gene score with only one degree of freedom has been suggested to handle the above mentioned limitation [8].

Gene score is defined as the sum of all the risk alleles of the selected variants present in each study participant. However, this approachfaces a problem when some of the SNPs are positively associated with the outcome of interest (i.e., increase the risk of disease), while some are protective (i.e., decrease the risk of disease). In order to overcome this limitation, SNP coding is adjusted before a gene score test in such a way that all the alleles are positively correlated with the outcome [9]. Another problem encountered in gene scoring is the use of information from all of the SNPs, although some SNPs have low and others have high effect sizes, resulting in the reduction in the study power. The current ways to deal with these issues include modified forward multiple regression (*MFMR, has higher power to detect weak genetic effects and has limited number of false positives),* Bonferroni correction (*used to counteract the problem ofmultiple comparisons, particularly when many SNPs are included simultaneously, the p-value used for statistical significance cutoff is 0.05/the number of the SNPs*), false discovery rate (FDR, *a method of conceptualizing the rate of type I errors in null hypothesis testing when conducting multiple comparisons* and randomization tests *(significance test that will have a false rejection rate always equal to the significance level of the test*) [10].

There has been scarce research on the obesitygenetics in Pakistan and most of it focussed monogenic forms. We have chosen those SNPs which are either candidate or GWAS hits for involvement in the energy regulation pathway. For the current investigation, ten SNPs were chosen because it is a pilot study and we were limited by the resources. It was taken care that the SNPs chosen had intermediate MAFs and they have previously been shown to predispose to obesity in other ethnicities. A gene score approach has not been tried for these SNPs in the Pakistani subjects. We are the first to use this approach to our ethnic group. We therefore aimed to look for any difference which use of a genetic risk score can make in comparison to the individual risk variants.

## Methods

### Study subjects

The study was a case control observational type and included 475 subjects (250 cases and 225 controls). Study subject recruitment was done from various cities of Punjab, Pakistan. The study subjects’ recruitment details, inclusion and exclusion criteria have been described elsewhere in detail [11]. The inclusion criteria were BMI and WHR cut offs defined for Asian populations previously (for obese cases: BMI > 23Kg/m^2^ as overweight and > 26Kg/m^2^, for controls: BMI < 23Kg/m^2^). Exclusion criteria for both cases and controls included pregnancy, presence of malignancies and recent infections. The study was approved by the institutional ethics committee (Ethical Committee, School of Biological Sciences, University of the Punjab, Pakistan), subjects gave a written informed consent and all procedures were carried out in compliance with the Helsinki declaration.

### Anthropometric measurements, blood sampling and biochemical analyses

The measurement of body weight (Kg), height (m), waist and hip circumference (cm) was according to standard procedures as described previously [12]. BMI (body mass index, Kg/m^2^) and WHR (waist to hip ratio) were calculated for each study subject. Blood samples were taken after 8-12 h fasting, half sample was used for DNA isolation while the rest half was used to obtain serum. Serum was separated by centrifuging gel vacutainers at 14,000 rpm for 10 min, collected in sterilized eppendorf and screened for any infectious agents (HBV, HCV, HIV). Any positive samples were discarded and safe samples were used for the lipid profile determination. Serum total cholesterol (TC), triglycerides (TG), high density lipoprotein cholesterol (HDL-C), and low density lipoprotein cholesterol (LDL-C) were measured using commercially available kits (Spectrum Diagnostics, Egypt). Epoch, Biotek microplate reader (Biotek instruments, Highland Park) was used for all optical density measurements.

### Genotyping

Genomic DNA was isolated from blood leukocytes using Wizard® Genomic DNA purification kit (Promega, USA). DNA was quantified using nanodrop (ND-8000, USA), and made to a 5 ng/μl concentration. The variants included the common SNPs in the genes involved in either the energy regulation (candidate) genes or GWAS implicated (non-candidate) genes (Additional file 1: Table S1). The genotyping methodologies for these SNPs were based on PCR-RFLP, tetra-ARMS or TaqMan methods (leptin (*LEP*) gene SNP G2548A, leptin receptor (*LEPR*) SNP Gln223Arg, and fatty acid binding protein 2 (*FABP2*) SNP Ala54Thr, were genotyped by PCR-RFLP method, the *FTO* gene SNP by tetra-ARMS PCR and rs3923113 near growth factor receptor bound protein (*GRB14*), rs16861329 in sialyltransferase 6 galactosidase 1 protein (*ST6GAL1*), rs1802295 in vacuolar protein sorting associated protein (*VPS26A*), rs7178572 in high mobility group protein 20 A (*HMG20A*), rs 2,028,299 in adaptor related protein complex (*AP3S2*) and rs4812829 in hepatocyte nuclear factor (*HNF4A*) by TaqMan allelic discrimination assay). The reaction mixture composition and PCR conditions have been described previously [11–14].

### Gene score (GS) calculation & statistical analysis

For the *GRB14* and *ST6GAL1* SNPs, the major alleles while for the rest the minor alleles were risk alleles. SPSS was used to construct the gene score of the included variants. The SNPs were coded as 0, 1, and 2 for presence of no, one and two risk alleles i.e., homozygous protective, heterozygous and homozygous risk genotype, respectively. A new variable named ‘Gene Score’ was computed in the SPSS by adding up the number of the risk alleles for all the SNPs in each subject (e.g., if a subject has the allele profile for all variants as 0, 0, 1, 2, 0, 1, 1, 2, 0, 1, 1, 2, and 0, the gene score would be 0 + 0 + 1 + 2 + 0 + 1 + 1 + 2 + 0 + 1 + 1 + 2 + 0 = 11). The trend of gene score in cases and controls was analyzed by a normal distribution curve and the effect on anthropometric and biochemical traits was checked using linear regression taking obesity or lipid traits as dependent and gene score as the independent variable. The analyses were adjusted for confounders including age, gender, socioeconomic status (SES), hypertensive, diabetic, CVD status, etc. The difference between mean gene score in cases and controls was checked by the independent sample *t*-test. Due to the inclusion of multiple SNPs, a corrected *p*-value (0.05/10 = 0.005) was used as a significance cutoff.

## Results

The study subject characteristics have been published previously (Table 1) [11]. The reference SNPs’ information including name, respective gene and the minor allele frequency in the cases and the controls is given in Additional file 1: Table 1. Table 1 showed that all the parameters except height differed significantly between the cases and the controls as tested by the independent sample *t*-test. The lipid profile parameters deviated from normal ranges (with TC, TG and LDL-c significantly increased and HDL-c significantly decreased) in the cases as compared to the controls. The genotyping call rates for all the SNPs were ~ 98%.

**Table 1:** Study Population Anthropometric and Biochemical characteristics [1]

Characteristics	Obese (*n* = 250)	Non Obese (*n* = 225)	*p*-value
Gender			
Males	139 (55.6%)	118 (52%)	–
Females	111 (44.4%)	107 (48%)	–
Family History	159 (63.6%)	19 (8.4%)	–
Age (years)	39.63 ± 15.19	37.78 ± 11.53	< 0.001
Weight (Kg)	95.56 ± 16.05	68.63 ± 10.23	< 0.001
Height (ft)	5.36 ± 0.43	5.4 ± 0.91	0.536
BMI(Kg/m2)	34.37 ± 6.08	22.67 ± 5.58	< 0.001
WC (cm)	99.55 ± 12.11	71.95 ± 8.65	< 0.001
HC (cm)	108.51 ± 12.56	78.23 ± 8.34	< 0.001
WHR (WC/HC)	1.00 ± 0.07	0.78 ± 0.01	< 0.001
FPG (mg/dL)	102.71 ± 14.12	89.56 ± 7.29	< 0.001
Total Cholesterol (TC) (mmol/L)	5.63 ± 0.90	4.11 ± 0.79	< 0.001
Triglycerides (TG) (mmol/L)	2.64 ± 0.83	2.12 ± 0.05	< 0.001
HDL-c (mmol /L)	1.11 ± 0.10	2.09 ± 0.44	< 0.001
LDL-c (mmol/L)	2.57 ± 0.56	2.02 ± 0.34	< 0.001
Gene Score	9.1 ± 2.26	8.35 ± 2.07	< 0.001
Minimum	3	0	
Maximum	20	13	

Legend: The table summarizes the general, anthropometric and biochemical characteristics of the study population. Values are indicated as mean ± SD. *BMI* Body mass index, *WC* Waist Circumference, *HC* Hip Circumference, *WHR* Waist to Hip Ratio, *FPG* Fasting Plasma Glucose, *HDL-c* High Density Lipoprotein cholesterol, *LDL-c* Low Density Lipoprotein cholesterol

### Gene score distribution in the cases and the controls

**Fig. 1:**
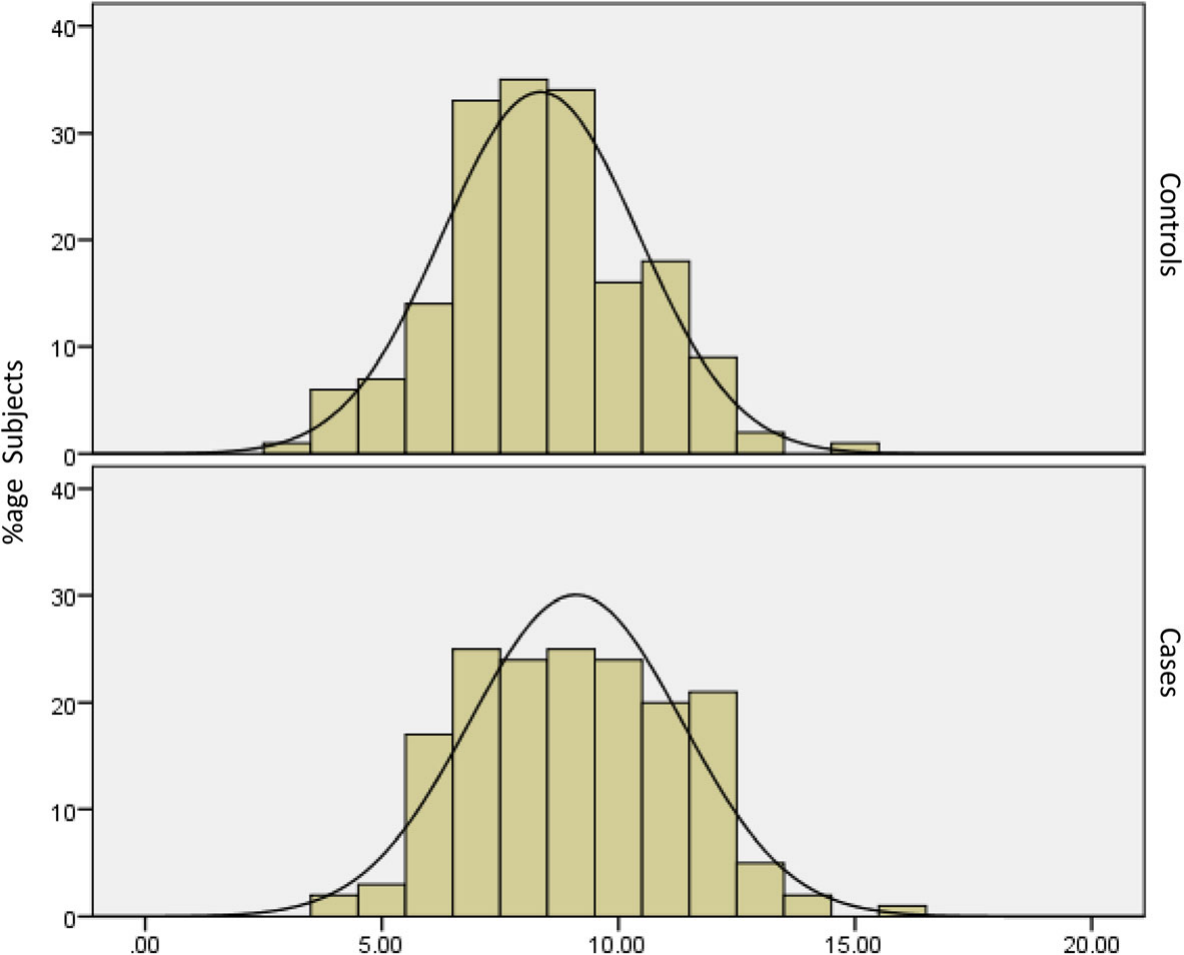
Histograms of Gene score in cases and controls. The histograms show the normal distribution of the risk allele count of the study participants, the top half for the controls and the bottom half for the cases. On the x-axis, gene score is plotted and on y-axis, frequency is mentioned. The bars show the respective frequency of study subjects in each group on that particular gene score

The comparison of the gene score between cases and controls is given in the Fig. 1. It shows that the curve is shifted towards right in the cases indicating that a greater number of individuals possessed a higher gene score as compared to the controls whereby the majority of the individuals had a lower number of risk alleles. The mean gene score of the participants is given in Table 1 and showed that in controls (8.35 ± 2.07) and cases (9.1 ± 2.26) was significantly different (*p* = 2 × 10^− 4^). As ten SNPs were included in the analysis, the maximum number of risk alleles an individual could possess is twenty. The descriptives of the gene score are summarized in Table 1.

### Comparison of the effect of gene score and individual variants on obesity

In order to check whether the use of the gene score approach improves the association of the genetic component to obesity as compared to the individual variants, we performed a linear regression analysis. The association of the individual variants and the gene score with the obesity showed that the individual variants had either marginal or no significant association with obesity, but the gene score was highly significantly associated with the obesity. The *p*-values in the table indicate the strength of association of the single SNPs and the gene score (Table 2).

**Table 2:** Comparison of Association of Individual SNPs and Gene Score with Obesity

Gene	SNP/GS	OR	*p-*value
*GRB14*	rs3923113	0.903	0.598
*ST6GAL1*	rs16861329	0.586	0.007
*VPS26A*	rs1802295	1.366	0.087
*AP3S2*	rs2028299	1.351	0.094
*HNF4A*	rs4812829	1.429	0.047
*HMG20A*	rs7178572	1.149	0.373
*FABP2*	Ala54Thr	1.379	0.045
*LEP*	G2548A	1.560	0.006
*LEPR*	Gln223Arg	1.379	0.045
*FTO*	rs1121980	1.346	0.089
Gene Score		1.175	0.0001

The OR for the individual SNPs and the *p*-values indicate a marginal or no association with the outcome, however, when a gene score is used, the association with obesity becomes significant

### The effect of the gene score on anthropometric parameters

**Fig. 2:**
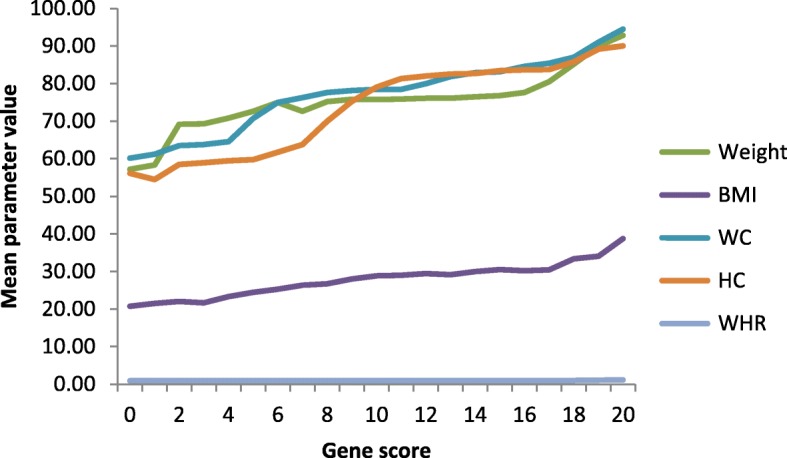
Effect of gene score on the anthropometric traits. The figure shows gene score on x-axis and mean values for the respective parameters on the y-axis. The slope of the lines indicate the trend of the parameter with increasing gene score

The effect of the gene score on anthropometric parameters is presented in Tables 3 and 4 , Table 3 summarizing the increase in the mean values of a parameter with increasing gene score and Table 4 showing the quantitative increase per increase of one risk allele. It is clear from Table 3 that the increase in the number of risk alleles (i.e. the gene score) increased the weight, BMI, WC, HC and WHR. This is further clarified in the Fig. 2 showing a graphical plot of the relationship of the gene score and the anthropometric traits. The effect of the gene score on the selected anthropometric traits appeared to be quantitative and is shown in Table 4. The beta effect means the per risk allele increase in a parameter and the *p*-value shows whether this increase is significant or not. It is clear that the per allele increase in weight, height, HC and WHR is insignificant while highly significant increase is observed for BMI and WC.

**Table 3:** Effect of Gene Score on the anthropometric traits

Gene Score	Weight (Kg)	BMI (Kg/m^2^)	WC (cm)	HC (cm)	WHR (WC/HC)
0	57.01 ± 8.11	21.75 ± 2.11	60.14 ± 5.01	56.14 ± 3.11	0.93 ± 0.02
1	57.93 ± 7.10	21.45 ± 2.62	65.09 ± 4.17	54.47 ± 2.64	0.95 ± 0.01
2	65.99 ± 5.11	21.96 ± 3.13	61.6 ± 2.11	57.43 ± 4.16	0.96 ± 0.01
3	68.46 ± 3.64	21.88 ± 3.07	63.45 ± 5.13	58.46 ± 5.10	0.95 ± 0.03
4	69.32 ± 5.01	22.64 ± 3.13	63.79 ± 4.36	58.92 ± 4.61	0.96 ± 0.01
5	70.78 ± 6.10	23.90 ± 2.63	64.56 ± 7.13	59.46 ± 4.16	0.95 ± 0.02
6	73.56 ± 8.14	24.62 ± 2.93	71.00 ± 6.13	60.98 ± 4.61	0.95 ± 0.01
7	74.00 ± 7.11	25.30 ± 3.13	75.00 ± 5.16	61.76 ± 4.35	0.96 ± 0.01
8	74.66 ± 8.03	26.12 ± 2.33	76.28 ± 4.95	63.75 ± 6.10	0.95 ± 0.01
9	75.16 ± 8.13	26.58 ± 1.98	77.63 ± 4.23	70.00 ± 2.64	0.96 ± 0.02
10	75.42 ± 8.09	26.99 ± 2.32	77.77 ± 8.64	72.40 ± 3.16	0.97 ± 0.01
11	75.75 ± 5.49	27.53 ± 2.06	78.48 ± 4.96	79.08 ± 3.69	0.96 ± 0.03
12	75.86 ± 5.64	28.94 ± 2.64	78.44 ± 8.06	81.30 ± 3.85	0.95 ± 0.01
13	76.45 ± 6.14	29.09 ± 2.16	78.91 ± 7.46	81.39 ± 4.06	0.97 ± 0.01
14	76.88 ± 6.06	29.55 ± 2.06	79.96 ± 5.09	82.00 ± 2.61	0.97 ± 0.01
15	76.92 ± 5.31	29.61 ± 2.43	81.85 ± 6.11	82.51 ± 3.94	0.98 ± 0.03
16	76.99 ± 5.14	29.97 ± 3.16	82.88 ± 6.46	82.65 ± 3.46	0.98 ± 0.06
17	77.58 ± 7.43	30.27 ± 1.37	84.58 ± 6.13	83.67 ± 2.63	0.99 ± 0.01
18	80.50 ± 4.16	30.60 ± 1.63	85.40 ± 5.14	83.73 ± 4.16	1.01 ± 0.02
19	84.60 ± 7.10	30.81 ± 3.43	87.00 ± 4.96	85.41 ± 3.65	1.01 ± 0.02
20	86.20 ± 4.22	31.83 ± 3.04	87.00 ± 7.06	85.75 ± 5.31	1.06 ± 0.03

The table shows the increase in the mean values of the tested parameters with increasing gene score indicating a quantitative effect. *BMI* Body mass index, *WC* Waist Circumference, *HC* Hip Circumference, *WHR* Waist to Hip Ratio

**Table 4:** Quantitative Effect of the gene score (risk allele) on selected traits, expressed as the beta effect (Standard error)

Parameter	β (SE)	*p*-value
Weight (Kg)	0.069 (0.005)	0.465
Height (ft)	0.222 (0.005)	0.051
BMI (Kg/m^2^)	0.622 (0.013)	0.001
WC (cm)	0.441 (0.005)	1.2 × 10^−3^
HC (cm)	0.111 (0.005)	0.111
WHR	0.010 (0.339)	0.106

The beta effect means the rise or fall in the selected trait per each risk allele increase. For example, for weight a beta effect of 0.069 means that with each risk allele increase, the body weight increases by 0.069Kg

### The effect of the gene score on biochemical parameters

The change in biochemical traits with increasing gene score is given in Table 5 and per risk allele increase in the mean values are shown in Table 6. The presence of an increasing number of risk alleles makes the lipid traits more dyslipidemic as indicated by the increase in the values of TC, TG and LDL and decrease in the concentration of HDLC with an increasing gene score in the Table 5. The quantitative effect of the gene score on the selected biochemical traits in Table 6 indicated a strongly significant increase in all the lipid parameters’ levels with the presence of each risk allele. A Bonferroni’s correction was made for analyses and a corrected *p*-value (0.005) was used for testing the significance of the association of the gene score due to inclusion of ten SNPs. Gene score appeared to be significantly associated with only BMI, WC and all lipid traits.

**Table 5:** Effect of Gene Score on the biochemical traits

Gene Score	Mean TC (mmol/L)	Mean TG (mmol/L)	Mean HDLC (mmol/L)	Mean LDLC (mmol/L)
0	4.05 ± 0.18	1.18 ± 0.07	2.18 ± 0.11	1.81 ± 0.09
1	4.25 ± 0.17	1.70 ± 0.07	1.99 ± 0.01	2.26 ± 0.01
2	4.40 ± 0.17	1.81 ± 0.09	1.99 ± 0.09	2.33 ± 0.09
3	4.45 ± 0.16	2.00 ± 0.06	1.95 ± 0.08	2.41 ± 0.08
4	4.59 ± 0.18	2.05 ± 0.07	1.89 ± 0.09	2.49 ± 0.08
5	4.65 ± 0.18	2.11 ± 0.06	1.81 ± 0.09	2.51 ± 0.12
6	4.71 ± 0.17	2.19 ± 0.09	1.79 ± 0.08	2.62 ± 0.09
7	4.79 ± 0.17	2.23 ± 0.13	1.61 ± 0.09	2.69 ± 0.11
8	4.81 ± 0.16	2.29 ± 0.11	1.55 ± 0.11	2.71 ± 0.09
9	4.81 ± 1.12	2.32 ± 0.04	1.51 ± 0.09	2.77 ± 0.11
10	4.85 ± 0.19	2.51 ± 0.012	1.49 ± 0.09	2.80 ± 0.13
11	4.89 ± 0.19	2.71 ± 0.09	1.42 ± 0.06	2.99 ± 0.08
12	5.00 ± 0.19	2.88 ± 0.09	1.39 ± 0.11	3.09 ± 0.08
13	5.05 ± 0.20	3.26 ± 0.08	1.37 ± 0.01	3.22 ± 0.08
14	5.11 ± 0.16	3.51 ± 0.08	1.31 ± 0.11	3.29 ± 0.08
15	5.16 ± 0.17	3.63 ± 0.08	1.25 ± 0.11	3.35 ± 0.09
16	5.19 ± 0.19	3.72 ± 0.08	1.18 ± 0.09	3.43 ± 0.13
17	5.23 ± 0.14	3.81 ± 0.16	1.15 ± 0.07	3.51 ± 0.11
18	5.31 ± 0.16	3.96 ± 0.11	1.13 ± 0.14	3.88 ± 0.09
19	5.43 ± 0.45	4.65 ± 0.06	1.01 ± 0.11	3.99 ± 0.11
20	5.61 ± 0.19	4.70 ± 0.04	0.81 ± 0.06	4.01 ± 0.11

The table shows the increase in the mean values of the tested parameters with increasing gene score indicating a quantitative effect. *TC* Total cholesterol, *TG* Triglycerides, *HDL-c* High Density Lipoprotein cholesterol, *LDL-c* Low Density Lipoprotein cholesterol

**Table 6:** Quantitative Effect of the gene score (risk allele) on biochemical traits, expressed as the beta effect (Standard error)

Parameter	β (SE)	*p*-value	*p-*value (adjusted for BMI)
TC (mmol/L)	0.168 (0.018)	3.01 × 10^−17^	1.12 × 10^−10^
TG (mmol/L)	0.090 (0.021)	2.71 × 10^−3^	1 × 10^−1^
HDLC (mmol/L)	−0.498 (0.039)	5.4 × 10^−43^	1.1 × 10^−13^
LDLC (mmol/L)	0.211 (0.029)	1.11 × 10^−21^	2.5 × 10^−6^

The beta effect means the rise or fall in the selected trait per each risk allele increase. For example, for TC, a beta effect of 0.168 means that with each risk allele increase, the serum total cholesterol increases by 0.168 mmol/L.

## Discussion

The use of a genetic risk score is not a completely new idea, it is being used in the risk scoring of heart diseases in addition to the conventional risk factors in many developed countries to decide about the appropriate therapeutic options [15] and a recent study in Pakistan also proposed the use of this approach [16]. It has been used for risk scoring of many polygenic disorders, however, its use is somewhat new for obesity. A recent study found significant association of genetic risk score for 32 loci with obesity in obese subjects with major depressive disorder [17], a 32-locus genetic risk score was also found to be statistically significant predictor of body mass index and obesity in White subjects from Atherosclerosis Risk in Communities (ARIC) cohort [18], whereas another study reported the association of gene score with serum triglyceride levels in morbidly obese Mexican subjects [19].

We used the gene score to study the combined effect of risk alleles on obesity. This is a robust approach, particularly when sample size is small, as it gives information regarding the additive effects of multiple variants in different genes in the same individual. The effect size assigned to each variant is independent of the effect estimated from the current small study such that the power problem is somewhat overcome. We selected ten variants from different genes that means an individual could have a maximum of twenty risk alleles. By using a gene score approach, we found that there is a significant difference in the mean gene scores between cases and controls which indicates the role of this index in the development of disease.

Among anthropometric traits, gene score appeared to be significantly associated with BMI and WC only. These are indices of central and abdominal obesity and association of the gene score with these parameters shows that the effect of each risk may be small on its own, but when combined can affect the overall fat distribution and disturb the fat metabolism resulting in an increase in BMI and WC. It is important to note, however, that although we could detect association with these two indices only, the trends of HC and WHR are also in the same direction. The lack of a statistically significant association may either be due to small sample size or the possibility that other variants which have not been included in the study may have influenced the effect of the SNPs.

It has been observed that many lipid/lipoprotein abnormalities are prevalent in obesity, such abnormalities are collectively termed as dyslipidemia, however, these dyslipidemias are often hyperlipidemia wherein majority lipids are shifted towards the upper limits of range or higher than the range. Obesity associated dyslipidemia is characterized by an increase in total cholesterol (TC), triglycerides (TG), low density lipoproteins (LDL-c), and decrease in high density lipoproteins (HDL-c), with TG and HDL being the most consistent and pronounced. One study considered fat distribution as an important factor for determining the differential distribution of TG, HDL and lipoproteins in both sexes and indicated lipid profile in obese persons as an important factor for progression to cardiovascular diseases [20–23]. We observed that the associations with the lipid traits became less significant when adjusted for BMI, while the association with TG was no longer significant. This showed that the associations were mediated somewhat by BMI. It is thus unclear whether lipid or obesity is causal for the others or the genes have pleiotropic effects on both traits.

The genetic contribution to obesity is well known in the present era and many remarkable achievements have been made in elucidating the role of genetics in the development of obesity. There is a long list of the candidate and non candidate genes known to be associated with obesity and we have comprehensively reviewed it previously [24]. We selected only a few from this list and from other sources retrieved from various search engines because of the resources available that supported the current analysis only. Because of this consideration, we tried to select a representative set of variants, from a number of genes so that this pilot study can provide us information about the significance to study role of these variants in context with obesity in the Pakistani subjects. The study has the limitation of relatively small sample size, inability to include more SNPs into analyses and different genotyping strategies for different SNPs. For the first two limitations, future studies should be planned to identify a panel of common variants associated with obesity in the Pakistani population. The limitation of genotyping techniques was relatively overcome by adopting a stringent control over genotyping call rate, the genotyping was repeated wherever a discrepancy was found. However, better results may have been obtained if all variants were genotyped by the same technique.

## Conclusion

As complex disorders are the result of interaction of a number of factors, special attention is needed to cope and treat these issues, one such major problem is ‘obesity’. In the era of current research, it’s not appropriate to rely only on the biochemical parameters of serum for treatment of problem after appearance of symptoms, rather use of information from different genes known to play a role in obesity in the concerned ethnic group should be used to calculate the ‘risk’ an individual possesses for the development of obesity in the future and treat the at risk individual so as to prevent the progression to obesity.

## Additional file

Additional file 1: **Table S1.** Reference information of the SNPs included in the study. LEP: Leptin, LEPR: Leptin Receptor, FABP2: Fatty Acid Binding Protein 2, FTO: Fat Mass and Obesity associated, GRB14: Growth factor receptor bound protein 14, ST6GAL1: Sialyltransferase 6 galactosidase 1 protein, VPS26A: Vacuolar protein sorting associated protein, HMG20A: High mobility group protein 20 A, AP3S2: Adaptor related protein complex, HNF4A: Hepatocyte nuclear factor 4 A. the Global MAF values are taken from SNPedia, while the *p*-value indicates the significance of difference between the GMAF and the MAF observed in the current study. (DOCX 31 kb)

## Abbreviation

BMI: Body Mass Index
DBP: Diastolic Blood Pressure
FABP2: Fatty Acid Binding Protein 2
FPG: Fasting Plasma Glucose
GRS: Genetic Risk Score
HC: Hip Circumference
HDL-c: High Density Lipoprotein cholesterol
HOMA-IR: Homeostasis Model Assesment of Insulin Resistance
LDL-c: Low Density Lipoprotein cholesterol
SBP: Systolic Blood Pressure
WC: Waist Circumference
WHO: World Health Organization
