# Supplementary material for: Use of a gene score of multiple low-modest effect size variants can predict the risk of obesity better than the individual SNPs
Source: Lipids Health Dis. 2018 Jul 18;17:155. doi: 10.1186/s12944-018-0806-5 (PMC6052513; doi:10.1186/s12944-018-0806-5)
Supplement: Supplementary file 1 — Table S1. Reference information of the SNPs included in the study. LEP: Leptin, LEPR: Leptin Receptor, FABP2: Fatty Acid Binding Protein 2, FTO: Fat Mass and Obesity associated, GRB14: Growth factor receptor bound protein 14, ST6GAL1: Sialyltransferase 6 galactosidase 1 protein, VPS26A: Vacuolar protein sorting associated protein, HMG20A: High mobility group protein 20 A, AP3S2: Adaptor related protein complex, HNF4A: Hepatocyte nuclear factor 4 A. the Global MAF values are taken from SNPedia, while the p-value indicates the significance of difference between the GMAF and the MAF observed in the current study. (DOCX 31 kb) [file 12944_2018_806_MOESM1_ESM.docx]

| SNP | Gene | Global MAF | MAF (%) | | *p*-value |
| --- | --- | --- | --- | --- | --- |
|  |  |  | Obese | Non Obese |  |
| G2548A | *LEP* | 43.34 | 42.8 | 30.1 | 0.115 |
| Gln223Arg | *LEPR* | 40.1 | 32.0 | 18.6 | <0.001 |
| Ala54Thr | *FABP2* | 25.21 | 42.4 | 33.1 | <0.046 |
| rs1121980 | *FTO* | 36.82 | 37.5 | 24.7 | 0.195 |
| rs3923113 | *GRB14* | 26.1 | 24.5 | 21.9 | 0.126 |
| rs16861329 | *ST6GAL1* | 25.0 | 23.2 | 17.8 | 0.065 |
| rs1802295 | *VPS26A* | 26.2 | 29.4 | 23 | 0.146 |
| rs7178572 | *HMG20A* | 52.0 | 52.9 | 52.1 | 0.665 |
| rs2028299 | *AP3S2* | 31.2 | 33.2 | 26.9 | 0.531 |
| rs4812829 | *HNF4A* | 29.1 | 31.0 | 24.8 | 0.098 |

Supplementary Table 1: Reference information of the SNPs included in the study. *LEP*: Leptin, *LEPR*: Leptin Receptor, *FABP2*: Fatty Acid Binding Protein 2, *FTO*: Fat Mass and Obesity associated, *GRB14*: Growth factor receptor bound protein 14, *ST6GAL1*: Sialyltransferase 6 galactosidase 1 protein, *VPS26A*: Vacuolar protein sorting associated protein, *HMG20A*: High mobility group protein 20 A, *AP3S2*: Adaptor related protein complex, *HNF4A*: Hepatocyte nuclear factor 4 A. the Global MAF values are taken from SNPedia, while the p-value indicates the significance of difference between the GMAF and the MAF observed in the current study.

| Gene symbol | Gene name | SNP ID | OR (95 % CI)* |
| --- | --- | --- | --- |
| *FTO* | Fat mass and obesity associated | rs9939609 | 1.31 (1.23–1.39) |
| Near *MC4R* | Melanocortin-4 receptor | rs17782313 | 1.12 (1.08–1.16) |
| Near *TMEM18* | Transmembrane protein 18 | rs7561317 | 1.2 (1.13–1.27) |
|  |  | rs6548238 | 1.19 (1.10–1.26) |
| *FAIM2* | Fas apoptotic inhibitory molecule 2 | rs7138803 | 1.14 (1.09–1.19) |
| Near *GNPDA2* | Glucosamine-6-phosphate deaminase 2 | rs10938397 | 1.12 (1.07–1.17) |
| *SEC16B* | *S. cerevisiae* Sec16 | rs10913469 | 1.11 (1.05–1.18) |
| *BDNF* | Homolog of brain-derived neurotrophic factor | rs925946 | 1.11 (1.05–1.16) |
| Near *ETV5* | Ets variant 5 | rs7647305 | 1.11 (1.05–1.17) |
| *SH2B1* | SH2B adaptor protein 1 | rs7498665 | 1.11 (1.06–1.17) |
| Near *NEGR1* | Neuronal growth regulator 1 | rs2568958 | 1.07 (1.02–1.12) |
| Near *KCTD15* | Potassium channel tetramerization domain containing 15 | rs29941 | 1.10 (1.04–1.15) |
|  |  | rs11084753 | 1.04 (0.98–1.10) |
| *MTCH2* | Mitochondrial carrier 2 | rs10838738 | 1.03 (0.98–1.08) |
| Near *PRKD1* | Protein kinase D1 | rs11847697 | 1.10 (1.03–1.17) |
| *SLC39A8* | Solute carrier family 39, member 8 | rs13107325 | 1.1 (1.05–1.15) |
| *TFAP2B* | Transcription factor AP-2 beta | rs987237 | 1.09 (1.05–1.12) |
| *QPCTL* | Glutaminyl-peptide cyclotransferase-like | rs2287019 | 1.09 (1.05–1.12) |
| *NRXN3* | Neurexin 3 | rs10150332 | 1.09 (1.05–1.12) |
| Near *GPRC5B* | G protein-coupled receptor, family C, group5, member B | rs12444979 | 1.08 (1.04-1.11) |
| Near *RBJ*- | *DNAJC27* DnaJ (Hsp40) homolog, subfamily C, member 27 | rs713586 | 1.07 (1.05–1.09) |
| *MAP2K5* | Mitogen-activated protein kinase 5 | rs2241423 | 1.07 (1.04–1.10) |
| Near *TMEM160* | Transmembrane protein 160 | rs3810291 | 1.06 (1.03–1.08) |
| Near *FANCL* | Fanconi anemia, complementation group L | rs887912 | 1.06 (1.03–1.08) |
| Near *FLJ35779*-*POC5* | Centriolar protein | rs2112347 | 1.05 (1.03–1.08) |
| Near*LRP1B* | Low density lipoprotein receptor-related protein 1B | rs2890652 | 1.05 (1.02–1.08) |
| *MTIF3* | Mitochondrial translational initiation factor 3 | rs4771122 | 1.05 (1.01–1.08) |
| *LRRN6C* | Leucine rich repeat neuronal 6C | rs10968576 | 1.04 (1.02–1.06) |
| *TNNI3 K* | Interacting kinase | rs1514175 | 1.04 (1.02–1.07) |
| *CADM2* | Cell adhesion molecule 2 | rs13078807 | 1.03 (1.00–1.06) |
| *NUDT3* | Nucleoside diphosphate linked moiety X type motif 3 | rs206936 | 1.03 (1.01–1.06) |
| Near *RPL27A* | Ribosomal protein L27a | rs4929949 | 1.03 (1.01–1.05) |
| Near *ZNF608* | Zinc finger protein 608 | rs4836133 | 1.03 (1.01–1.05) |
| Near *PTBP2* | Polypyrimidine tract binding protein 2 | rs1555543 | 1.02 (0.99–1.04) |
| *GNAT2* | Guanine nucleotide binding protein (G protein) alpha transducing activity | rs17024258 | 1.27 (*p* = 0.02) |
| *HS6ST3* | Heparin sulphate 6-O-sulfotransferase 3 | rs7989336 | 1.09 (*p* = 0.0001) |
| *HNF4G* | Hepatocyte nuclear factor 4, gamma | rs4735692 | 1.09 (*p* = 1.97 × 10^−5^) |
| *RPTOR* | Regulatory associated protein of MTOR, complex 1 | rs7503807 | 1.08 (*p* = 7.07x10-5) |
| *MRPS33P4* | Mitochondrial ribosomal protein S33 pseudogene 4 | rs13041126 | 1.08 (*p* = 0.001) |
| *ZZZ3* | Zinc finger, ZZ-type containing 3 | rs17381664 | 1.08 (*p* = 0.001) |
| *ADCY9* | Adenylate cyclise 9 | rs2531995 | 1.06 (*p* = 0.01) |

Supplementary Table 2: An overview of the loci recently identified to be associated with obesity [[1](#_ENREF_1)]. * some studies did not report CI but the p-values. SNP ID: Single Nucleotide Polymorphism identity, OR: Odds Ratio, CI: Confidence Interval

1. Albuquerque D, Stice E, Rodríguez-López R, Manco L, Nóbrega C: Current review of genetics of human obesity: from molecular mechanisms to an evolutionary perspective. *Molecular Genetics and Genomics* 2015, 290**:**1191-1221.
